# Assessing empowerment as multidimensional outcome of a patient education program for adolescents with chronic conditions: A latent difference score model

**DOI:** 10.1371/journal.pone.0230659

**Published:** 2020-04-21

**Authors:** Henriette Markwart, Franziska Bomba, Ingo Menrath, Katja Brenk-Franz, Gundula Ernst, Ute Thyen, Andrea Hildebrandt, Silke Schmidt

**Affiliations:** 1 Department of Health and Prevention, University of Greifswald, Greifswald, Germany; 2 Department of Pediatrics and Adolescent Medicine, University of Luebeck, Luebeck, Germany; 3 Department of Psychosocial Medicine and Psychotherapy, Jena University Hospital, Jena, Germany; 4 Department of Medical Psychology; Medical School Hannover, Hannover, Germany; 5 Department of Psychological Methods and Statistics, Carl von Ossietzky University Oldenburg, Oldenburg, Germany; Fordham University, UNITED STATES

## Abstract

**Objectives:**

The current study aims to examine the construct of empowerment in the context of a transition education program. Patient education programs strive to empower adolescents with chronic conditions to take responsibility for their own health care to manage their transition from pediatric to adult medicine. Our study aimed to identify the core components of patient empowerment and examined whether its components are responsive to a patient education program.

**Methods:**

Data was collected in two longitudinal studies involving *N* = 723 adolescents with chronic conditions. We used Latent Difference Score models (LDSm) of: 1) self-efficacy (GSE), 2) transition competence (TCS), and 3) patient activation (PAM) to quantify the latent variable of patient empowerment (PE). Additionally, the LDSm were extended to analyze the effects of group affiliation (intervention vs. control) and participants’ age on empowerment.

**Results:**

PE was identifiable by the three components. The intervention group developed significantly higher scores of PE compared to the control group. Age (13–21 years) did not moderate the relation between group affiliation and PE.

**Conclusions:**

We quantified PE successfully using a psychometric modeling of change. Patient empowerment is measureable and utilizable in the specific context of transition of adolescents with chronic conditions.

## Introduction

*Transition* is defined as the purposeful, planned movement from pediatric to adult medicine, which includes the process of taking responsibility for the management of one’s own chronic condition. Adolescents with chronic conditions are more likely to show maladjustments and achieve fewer milestones of personal development when compared to their healthy peers [1–3]. Patient education programs have proved to be effective in supporting adolescents with chronic conditions who are managing their own transition [3,4]. It is, however, still unclear at which age an adolescent should be included in an education program to positively influence the individuals’ development and to enable a successful transition. Furthermore, chronological age is a much investigated criterion regarding transition, but findings are contradictory [5,6]. In a systematic review of the research on transition, the optimal age range of transfer in 14 of the 15 papers was 18–19 years [6]. Furthermore, the literature reports different findings on delaying the transfer to an older age. In the quantitative studies, broad support was found for a delayed age of transfer, but in the qualitative research, only one study reported supportive evidence of such a delay [6]. Moreover, the preferred timing is on the one hand related to chronological age [7,8], but on the other hand, studies have shown that the timing of transition should be based on the level of maturity and responsibility, and not on chronological age [9,10]. Nevertheless, adolescents with chronic conditions should receive an age-appropriate health care tailored to their specific needs [11]. In sum, the optimal age of transition is still debated and thus requires further investigation in transition research.

In the last decades, patient empowerment has become an important construct in health care. There is a widespread belief that patients should be empowered, i.e., encouraged to take an active role in affairs of their own health and health care [12,13]. According to Fumagalli et al. [12], empowerment encompasses the acquisition of motivation and abilities which enable a patient to actively participate in health-related decision-making. Consequently, empowerment creates the opportunity for a higher level of self-determination in patients’ relationships with professionals. Zimmerman [14] defines empowerment as a multidimensional construct consisting of three components: an intrapersonal (feeling & awareness), interactional (knowledge & skills) and behavioral component (action). According to Zimmerman [14], empowerment is reached if a person “believes that he or she has the capability to influence a given context (intrapersonal component), understands how the system works in that context (interactional component), and engages in behaviors to exert control in the context (behavioral component)” (p. 590). However, there is still a lack of consensus in the conceptualization of the *patient* empowerment term and its operationalization strongly depends on the context of the study [3,12,13,15].

To represent the three components of Zimmerman [14] regarding the empowerment of a patient in the field of health care and transition, we chose the following three constructs: 1) self-efficacy, 2) transition competence, and 3) patient activation. *Self-efficacy* can be used to operationalize the intrapersonal component, since it is defined as the belief that one can perform a novel or difficult task, or cope with adversity [16]. *Transition competence* captures self-perceived knowledge, skills, abilities of self-management, and competences regarding the preparation and the progress of transition and can be regarded as the interactional component. *Patient activation* describes patients’ engagement in their own health care and related to the behavioral component. In most of the studies about empowerment, the components were investigated separately to show differences regarding the level of empowerment in subjects [17]. This is in contrast to theoretical papers defining empowerment as a multidimensional construct with multiple components that are linked to each other [12,14]. This definition requires an investigation of patient empowerment with a more comprehensive approach, such as structural equation modeling, to satisfy the multidimensional concept.

The aim of the current study is to show the enhancement of patient empowerment through a patient education program with a comprehensive approach, and to investigate an operationalization based on Zimmerman’s model of empowerment in the context of patient education programs [14]. The patient education program was intended to strengthen the preparedness of adolescents with chronic conditions in the phase of transition to be responsible for and self-manage their condition. We also investigated *patient’s age* as a possible moderating factor on the effectiveness of the patient education program. To identify all of the proposed components, we applied a measurement model that was not only based on an established conceptual theory of patient empowerment, but also on empirically validated measures using structural equation modeling (SEM) of change, specifically Latent Difference Score modeling (LDSm; e.g., [18]).

## Materials and methods

### Sample

Two cooperating longitudinal multicenter studies were conducted to evaluate the effectiveness of the newly established patient education program with a controlled trial [4,19–21]. The first study was conducted in 11 pediatric outpatient clinics and one rehabilitation center in Germany [22]. The second study recruited the participants from 29 pediatric outpatient clinics, two hospitals and two self-help groups, also in Germany. In the final data, we included only the outpatient clinics to ensure the comparability of the data.

Data were collected in 40 health centers in Germany from September 2012 to December 2014. Each clinic provided a special consultation hour for adolescents with chronic conditions in an ambulant setting. Adolescents were recruited by their pediatricians using personal, mail and phone contact. The data were measured at a baseline (T0) and at a follow-up (T1) carried out 1–6 months later. Each participant and their parents or legal guardians gave informed consent. Eligible participants were adolescents aged from 13 to 25 years who were diagnosed with a chronic condition according to the ICD-10, for example type 1 diabetes, chronic inflammatory bowel disease, or cystic fibrosis, and who were still being treated in pediatric medicine prior to their transfer to adult medicine. Patients were assigned to groups based on their availability at predefined workshop dates; therefore, group affiliation was defined as an intervention group or control group. The control group did not take part in the patient education program, but filled out the questionnaires at the same measure points as the intervention group, a baseline (T0) and a follow-up (T1). In order to achieve an adequate participants-to-parameter ratio [23], we merged the two studies into one data file that is analyzed in the present article. In total, *N* = 723 adolescents fully completed both questionnaires and were included in the analysis. Their average age was *M*_*age*_ = 16.98 (*SD*_*age*_ = 1.64). Further demographic details are provided in Table 1. The numbers of male and female participants were about equal (49.1% and 50.9%, resp.), most of whom lived with their parents (*n* = 676, 93.8%). 1.8% indicated to live alone, while the remaining 4.4% lived in a different setting (e.g., living community or residential home). 56.3% of the sample participated in the patient education program and will be referred to here as the *intervention group*. After completing the questionnaires, all participants either received a EUR 20 voucher or they could take part in a prize draw to win an online voucher.

**Table 1:** Sociodemographic data of control group, intervention group, and total sample of latent difference score model of empowerment.

	Total Sample		
	Control Group (n = 316)	Intervention Group (n = 407)	Total (n = 723)
**Age**			
M	17.09	16.90	16.98
SD	1.70	1.51	1.60
Range	13–14	14–24	13–24
	Count (%)	Count (%)	Count (%)
**Sex**	(n = 316)	(n = 407)	(n = 723)
Male	162 (51.3)	193 (47.4)	355 (49.1)
Female	154 (48.7)	214 (52.6)	368 (50.9)
**Education**	(n = 314)	(n = 400)	(n = 714)
<12 years	130 (41.4)	155 (38.8)	285 (39.9)
≥12 years	164 (52.2)	226 (56.5)	390 (54.6)
others	20 (6.4)	19 (4.8)	39 (5.5)

### Patient education program

We aimed to show the enhancement of patient empowerment through the participation in a patient education program strengthening the preparedness of young people to be responsible for and self-manage their condition. The program intended to facilitate the exchange of practical knowledge and information, as well as interactions among participants, initiated through various interactive group methods. The patient education program was designed to contribute to the level of patient empowerment in the context of transition [3,12,13,15].

The program was devised as a group training for at least four adolescents on two consecutive days. It consisted of nine modules covering the following topics: transfer to adult medicine, orientation in the health system, future planning and occupation/career, separation from parents, communication about illness with peers and parents, stress management, and activation of resources. Each module had a duration of 60–90 minutes. The content of the curriculum was mainly generic, but some modules included condition-specific aspects. The program was conducted by a psychologist and a pediatrician; in addition, a doctor for adult medicine and a young adult with the same chronic condition like the participants of the intervention were invited as experts. It was executed in a workshop setting to initiate group interactions and promote the exchange of knowledge and information between the participants, following the guiding principles of Zimmerman’s empowerment definition [14,19]. Since the aim of the patient education program is to enable adolescents with chronic conditions to learn about their transition, to develop skills to manage this process, and to promote their ability and motivation to manage their chronic condition themselves, the participants had the opportunity to address their concerns, fears and problems, such as their fears of the future, or the everyday stress of managing their chronic condition [19].

Examples from the workshop include: participants role played getting prepared for the first consultations with their new doctor, they learnt to understand their laboratory results and technical terms (e.g. anatomy), or they drew a portrait of their “dream doctor”. In small groups, participants were invited to discuss changes regarding the transfer to adult medicine, and to articulate associated opportunities and risks. They were provided with information on contraception, drugs, alcohol and family planning that was relevant to their chronic conditions, and they discussed the usefulness of some websites and other sources of information. They furthermore became aware of their disease-related rights and obligations during their future studies and occupations, and they learnt how to obtain information from a variety of health care services and institutions, such as conducting phone calls with their health insurance. Lastly, the participants of the group intervention met with another young adult who suffered from the same chronic condition but experienced the transition already.

A more detailed description of the program’s content and development is provided in [19–21].

### Study measures

We carefully selected three components according to Zimmerman’s definition of empowerment [14] and placed them in the specific context of health care: self-efficacy representing the intrapersonal component, transition competence representing the interactional component, and patient activation corresponding to the behavioral component [12,14].

#### General Self-Efficacy scale (GSE)

The general self-efficacy scale measures perceived self-efficacy by means of 10 items (e.g., “I am confident that I could deal efficiently with unexpected events”), scoring from 1 (not at all true) to 4 (exactly true). A global score represents the self-perceived competence of a person to deal with daily hassles and stressful life time events. Thus, a higher score means a higher general self-efficacy. The reported Cronbach’s alpha in German samples ranged from .80 to .90 [16].

#### Transition Competence Scale (TCS)

The health-related transition competence scale measures the self-perceived knowledge, skills, abilities of self-management, and competences in the preparation of and the progress in transition, by means of 10 items (e.g., “I know the difference between adult health care and child health care”). The patient activation is quantified by a sum score ranging from 10 to 40 and can be transformed to a scale with a range between 0 and 100. The response categories of the 4-point Likert scale are between1 (strongly disagree) and 4 (strongly agree), where a higher score represents a higher self-perceived competence. The instrument contains three subscales: (1) work-related preparedness, (2) condition-related knowledge, and (3) health-related competence. The three subscales can be combined to a global score which has a reported Cronbach’s alpha of .81 and a discrimination index ranging from .42-.62 [24]. The global score correlates positively with the three subscales: (1) r = .79, (2) r = .79 and (3) r = .81, as reported by Hermann-Garitz et al. [24].

#### Patient Activation Measure (PAM)

The patient activation has previously been operationalized through the Patient Activation Measurement adapted for adolescents with chronic conditions [25]). This instrument consists of 13 items with response categories located on a 4-point Likert scale ranging from 1 (strongly disagree) to 4 (strongly agree). The PAM^®^ 13 for adolescents is an adapted version of the Patient Activation Measure (PAM-13^©^) [17], which measures the patients’ engagement in their own health care (e.g., “I have been able to adapt my daily routine according to my health condition”; “Taking an active role in my own health care is the most important factor in determining my health and ability to function”). The patient activation is quantified by a sum score ranging from 13 to 52, where higher scores represent higher levels of patient activation. The reported internal consistency of the previous study is satisfactory with a Cronbach’s alpha coefficient of α = .79. The retest reliability was reasonably high, with *r*_tt_ = .68 [25].

*Data preprocessing and handling of missing values*. The two cooperated studies were tested for their compatibility and the feasibility of merging the two data sets. Differences in the distribution of sex, age, education, and the mean scores of PAM and TCS in the two studies were investigated. Since the pre-analysis showed no differences, the items of GSE, TCS, and PAM were parceled, in order to reduce psychometric problems associated with modeling Likert scales with structural equations [26]. For the measurement of each scale, three parcels were composed by a random assignment of an equal number of items. This is a common method of parceling in the literature, given the unidimensionality of the scale [26]. There were missing data on the second time point of measurement in 55.3% of the total sample. However, to avoid information loss and to achieve a high test power, cases with missing data were not excluded from the analyses.

The estimation of the LDS measurement model of the empowerment components GSE only consisted of *N* = 323. The reason is that one of the multicenter samples was not asked to fill out the GSE, in order to minimize the demands the participants were exposed to. We handled missing values within the model of analysis by using the full information maximum likelihood (FIML) estimation procedure provided by the software Mplus [27]. FIML has the advantage that it will not exclude incomplete data patterns, but instead use all the available information for estimating the parameter of a postulated SEM.

### Data analysis

Latent difference score models (LDSm; [18]) were used to estimate the latent measurement error-free change (difference scores) observed at the level of manifest variables (using GSE, TCS, PAM parcels) at two time points: T0 (before the intervention) and T1 (after the intervention). There were three steps in the analysis: first, the latent difference score models of GSE, TCS, and PAM were separately estimated and these measurement models were used to identify patient empowerment, defined as a higher order factor determined by the estimated latent change factors for the three components. This is necessary because empowerment can only be assumed if there is a significant change in all three domains. Second, to test the intervention success, the latent variable *patient empowerment* was regressed into a dummy variable coding the group affiliation of participants (0 –control group, 1 –intervention group), because the intervention group was expected to develop a higher level of empowerment after 1–6 months compared with the control group. Third, we investigated whether age influenced the association between group affiliation and patient empowerment. This step was necessary to determine whether age represents a necessary factor to be included the patient education program.

The overall fit expressed by the χ^2^-Test and the following three alternative fit criteria was considered to evaluate the models: the Root Mean Square Error of Approximation (RMSEA), which should be lower than .08, the Comparative Fit Index (CFI), which should exceed.95 and the Standardized Root Mean Residual (SRMR), which is expected to be lower than .08 [28]. All reported analyses were conducted with Mplus 7.31 [27]. Because of the sizeable sample of *N* = 723 and the number of the tested models, we only considered *p*-values < .01 to be substantial.

**Fig 1:**
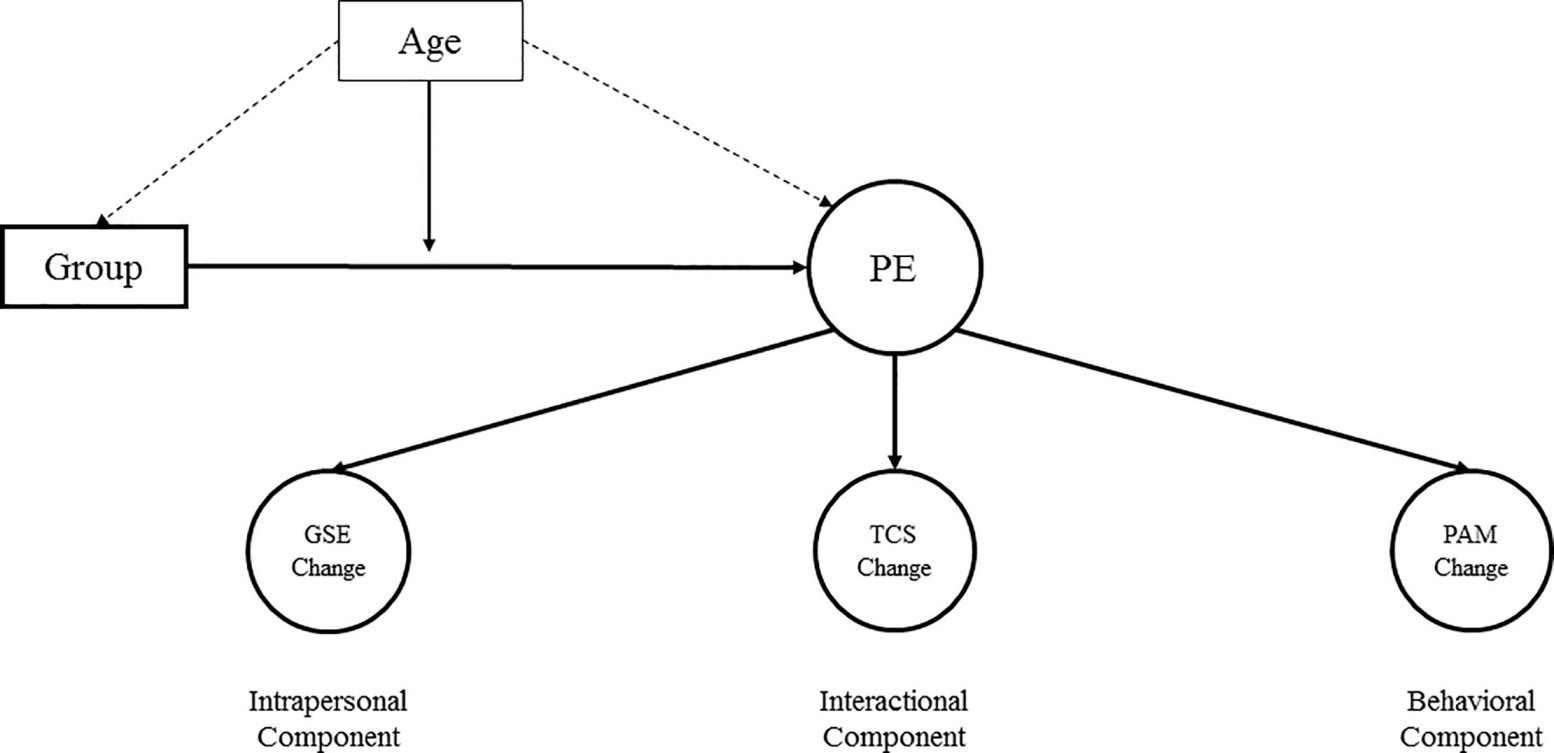
Theoretical model. Identification and Testing of Patient Empowerment PE = Patient Empowerment, GSE = General Self-Efficacy, TCS = Transition Competence Scale, PAM = Patient Activation Measurement–German Youth Version, Group = Group Affiliation, Age = age of the participants; model based on Zimmerman (1995).

*Latent difference score modeling* (LDSm) was proposed by McArdle [18] to measure the absolute change at the level of a latent variable. Since then, LDSm has been mainly applied in cases of two measurement time-points. Thus, latent difference scores represent interindividual differences in absolute intraindividual changes, adjusted for measurement errors. In order to estimate the changes, the measured latent variable at the second time-point is deconstructed into two parts: the estimated value of the latent variable at the first measurement time-point, and the change between the two measurement occasions. Thus, because patient empowerment has been defined in the literature [12,14] as a change score, we estimated the changes at the level of GSE, TCS, and PAM scores and modeled patient empowerment as a higher order factor indicated by these change scores (see Fig 1 representing the model structure). Such a latent variable modeling approach is completely novel in the literature on patient empowerment, while it is strongly required by usual theorizing (see above).

## Results

### Measurement models of change for general self-efficacy, transition competence and patient activation

**Fig 2:**
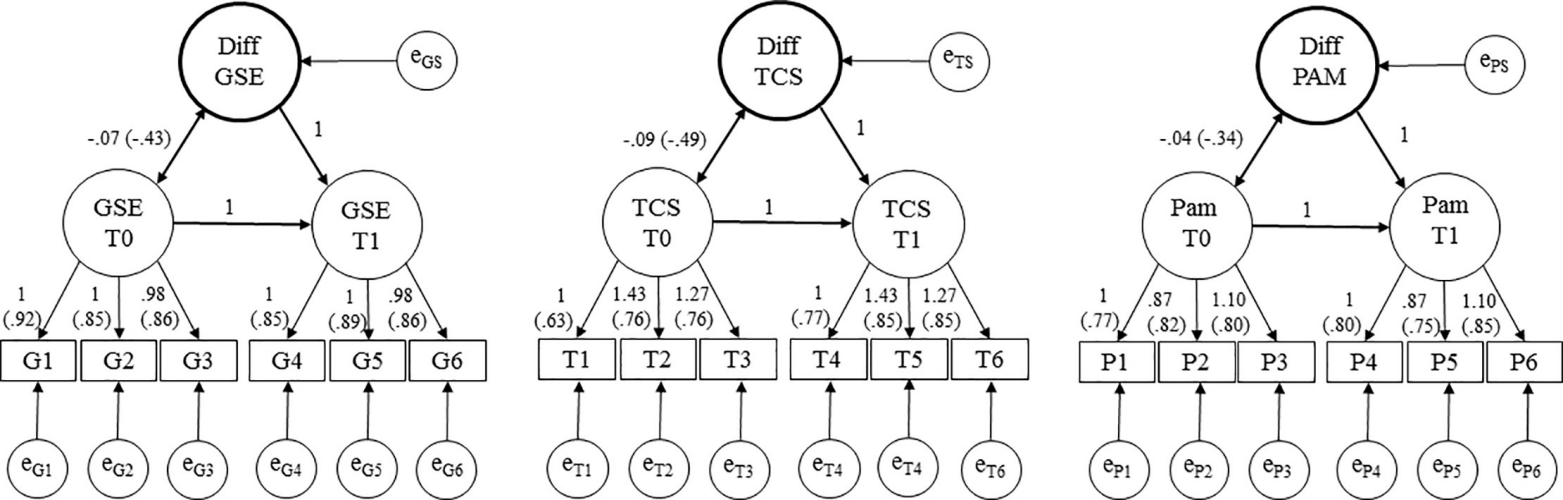
LDSm of self-efficacy, transition competence and patient activation. Unstandardized factor loadings (Standardized factor loadings). All results p < .001. LDSm = Latent Difference Score model, Latent GSE = General Self-Efficacy, TCS = Transition Competence Scale, PAM = Patient Activation Measurement–German Youth Version. Diff GSE = latent Difference of General Self-Efficacy, Diff TCS = latent Difference of Transition Competence Scale, Diff PAM = latent Difference of Patient Activation Measure.

In the first step, we estimated three separate measurement models of change for General Self-Efficacy (GSE), Transition Competence Scale (TCS) and Patient Activation Measure (PAM). The fit statistics for the three LDS models of GSE, TCS, and PAM suggested good to acceptable descriptions of the observed data. The TCS model (χ^2^(*df* = 9) = 20.24, *p* < 0.05, CFI = 0.99, RMSEA = 0.04, SRMR = 0.03) and the PAM model (χ^2^(*df* = 9) = 4.90, *p* > 0.05, CFI = 1.00, RMSEA = 0.00, SRMR = 0.01) had an excellent global fit. The GSE model had an acceptable global fit (χ^2^(*df* = 9) = 29.62, *p* < 0.01, CFI = 0.98, RMSEA = 0.08, SRMR = 0.15). These three measurement models revealed factor loadings, which were all statistically significant and homogeneous in their magnitude (shown in Fig 2). We thus considered them in the next step of analysis, which aimed to estimate the hierarchical model of patient empowerment. Importantly, in all three models, the latent difference score which should identify patient empowerment revealed significant interindividual variance (*M*_*GSE_Diff*_ = 0.07, *p* < 0.01, *σ*^*2*^_*GSE_Diff*_ = 0.14, *p* < 0.01; *M*_*TCS_Diff*_ = 0.43, *p* < 0.01, *σ*^*2*^_*TCS_Diff*_ = 0.20, *p* < 0.01; *M*_*PAM_Diff*_ = 0.11, *p* < 0.01, *σ*^*2*^_*PAM_Diff*_ = 0.08, *p* < 0.01).

### Hierarchical structural model of patient empowerment

**Fig 3:**
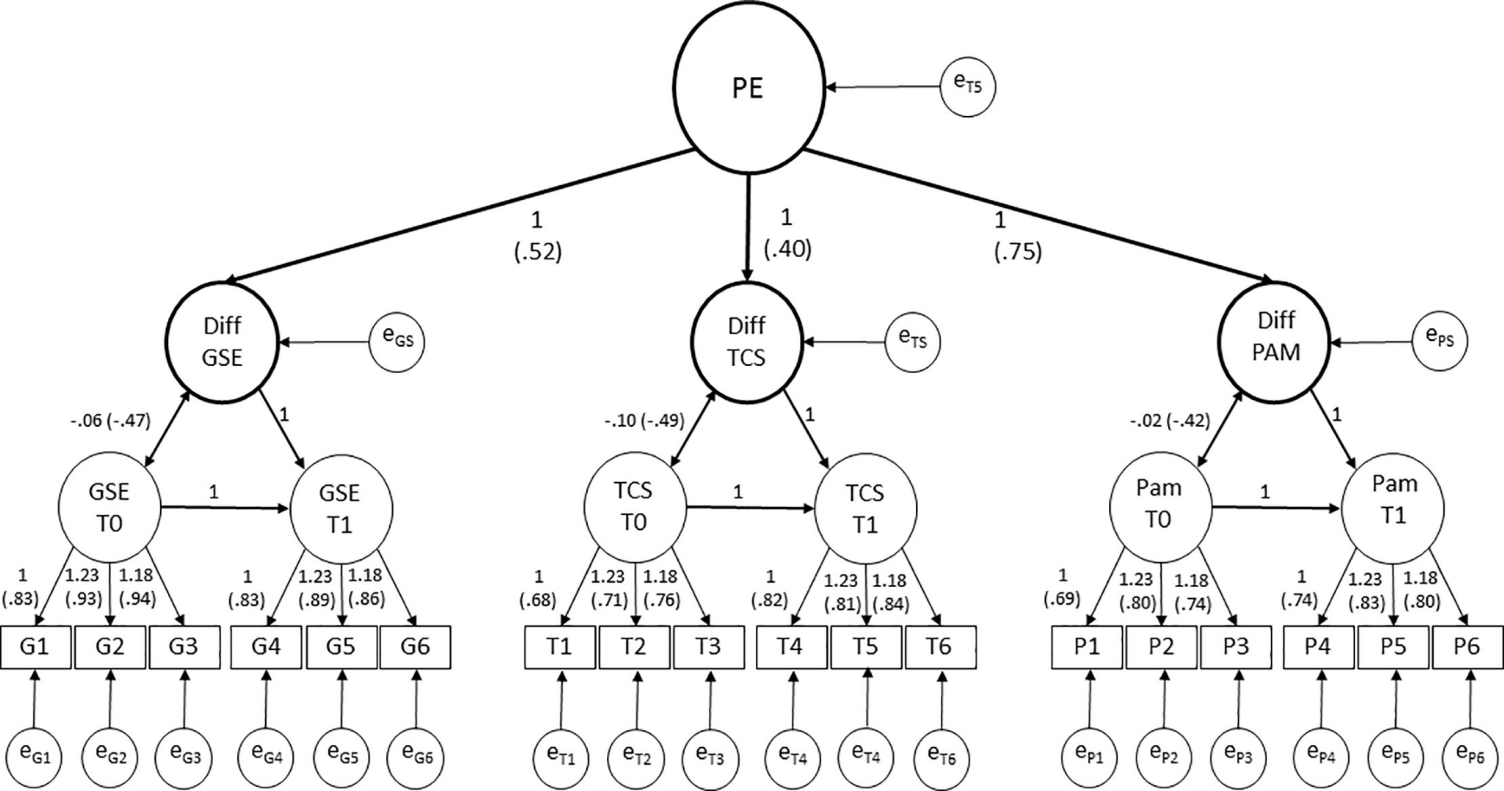
Hierarchical model. Identification of Patient Empowerment through Self-Efficacy, Transition Competence and Patient Activation Unstandardized factor loadings (Standardized factor loadings). All results p < .001. PE = Patient Empowerment, GSE = General Self-Efficacy, TCS = Transition Competence Scale, PAM = Patient Activation Measurement–German Youth Version. Diff GSE = latent Difference of General Self-Efficacy, Diff TCS = latent Difference of Transition Competence Scale, Diff PAM = latent Difference of Patient Activation Measure.

In the second step, a second order latent variable was estimated by the shared variance of the latent difference scores estimated for GSE, TCS, and PAM. The second-order latent variable labeled *patient empowerment* (PE) (χ^2^(*df* = 134) = 387.90, *p* < 0.01, CFI = 0.95, RMSEA = 0.051, SRMR = 0.12) was successfully identified through the latent difference scores of GSE with a loading on the second order factor PE of 0.52, (*p* < 0.01), TCS with a loading of 0.40 (*p* < 0.01), and PAM with a loading of 0.75, (*p* < 0.01). Moreover, the variance of PE was significantly different from zero (*σ*^*2*^_*PE*_ = 0.03, *p* < 0.01, with *M*_*PE*_ fixed to 0 for scaling purpose). Thus, the hierarchical model to identify PE is feasible to test the intervention success by adding a dummy variable predicting PE (see Fig 3).

### Influence of the patient educational program

**Fig 4:**
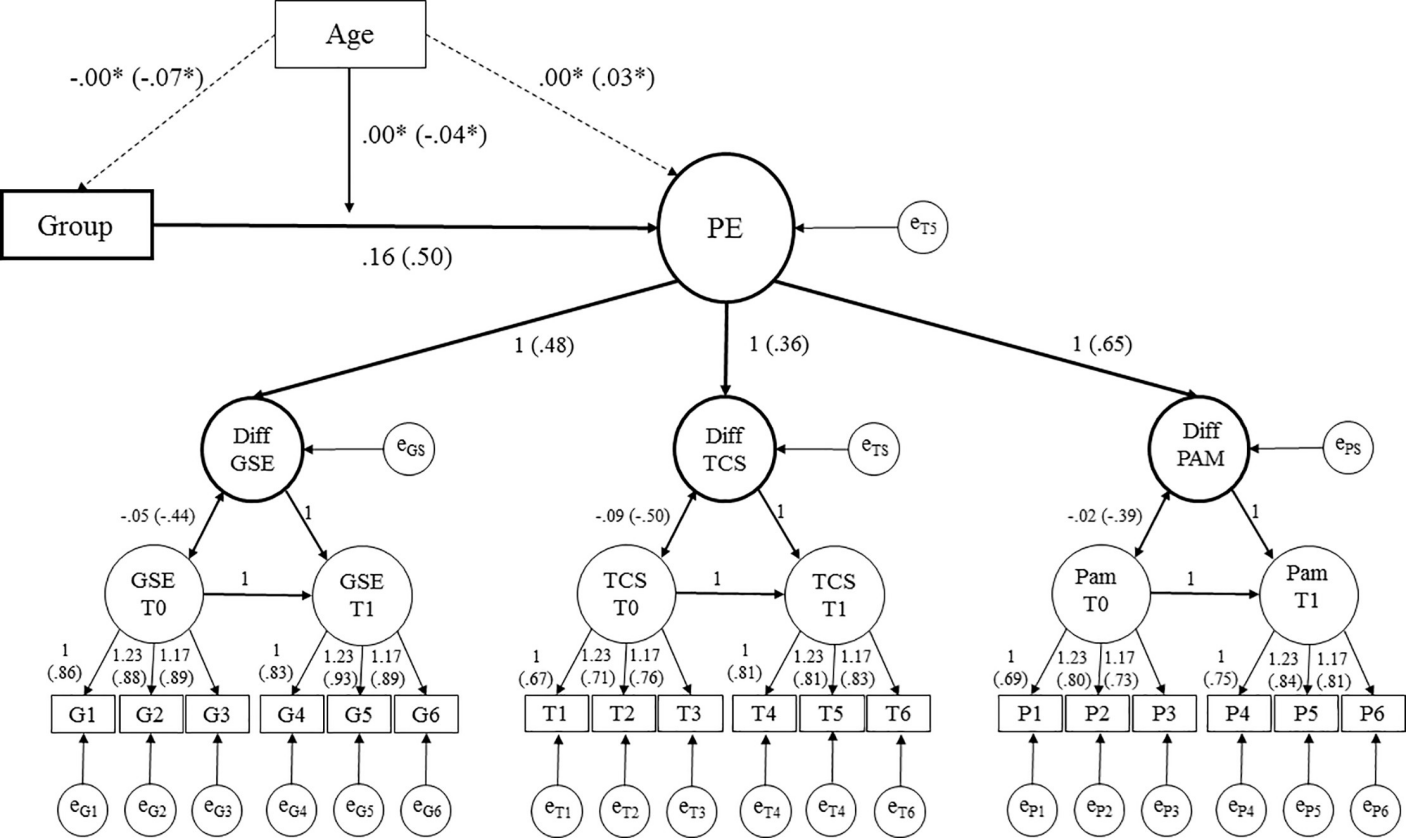
Extended hierarchical model. Patient Empowerment identified through Self-Efficacy, Transition Competence and Patient Activation influenced by group affiliation and age Unstandardized factor loadings (Standardized factor loadings). All results p < .001. * p>.05. PE = Patient Empowerment, GSE = General Self-Efficacy, TCS = Transition Competence Scale, PAM = Patient Activation Measurement–German Youth Version. Diff GSE = latent Difference of General Self-Efficacy, Diff TCS = latent Difference of Transition Competence Scale, Diff PAM = latent Difference of Patient Activation Measure. Group = Group Affiliation. Age = z-standardized age of the participants.

In the third step, the hierarchical model of PE was extended through the dummy variable coding group affiliation. The extended model had an acceptable fit (χ^2^(*df* = 157) = 539.07, *p* < 0.01, CFI = 0.92, RMSEA = 0.06, SRMR = 0.13). The regression weight of group affiliation (intervention/control) on PE was substantial, with a non-standardized regression weight of 0.16 (*p* < 0.01; see Fig 4) corresponding to a standardized regression weight of 0.50. Thus, we conclude that the intervention has a significant effect on the development of patient empowerment.

### Influence of age

The age of the participants was centered to the mean and included as a manifest variable in the hierarchical model to investigate the association between age and patient empowerment through the patient education program. Age was introduced as a moderator of the association of group affiliation and patient empowerment. The age-moderated LDSm showed an acceptable fit (χ^2^(*df* = 192) = 587.46, *p* < .001, CFI = 0.92, RMSEA = 0.05, SRMR = 0.12). The standardized moderation effect of age on the relationship between intervention and PE was not significant (-.04, *p* = 0.66; see Fig 4 for details).

## Discussion

This study has demonstrated the feasibility of measuring the construct of patient empowerment through a patient education program by using a comprehensive approach including the three components of Zimmerman [14] in the specific context of health care. Additionally, the influence of group affiliation (control group vs. intervention group) and age could be identified.

The predicted model of patient empowerment (PE), which was examined through a patient education transition program, was identified and analyzed. Based on the definition of empowerment, three components were used to identify PE as a multidimensional construct: 1) self-efficacy (GSE), 2) transition competence (TCS), and 3) patient activation (PAM). The three components deducted from Zimmerman [14] in the context of transition demonstrated that the predicted model of PE was identifiable. The latent difference scores of TCS, GSE, and PAM loaded significantly positive on the latent variable PE. The higher the latent difference of TCS, GSE and PAM, the higher the PE. Consequently, the three components could be used as indicators for PE. These findings are in line with Aujoulat et al. [15], thus suggesting once more that self-efficacy, self-management and patient activation are frequently anticipated outcomes in association with empowerment.

There is no consensus on the definition of patient empowerment and the theoretical foundation is often missing [30]. Rappaport and Seidman [29] stress that in the operationalization of empowerment, it is crucial to consider the setting and the context. In our study, we based our operationalization on a clear theoretical foundation as well as we considered the special setting and context of transition.

Furthermore, the interpretation of empowerment depends on the context of the measurement [14,29,30,31]. Furthermore, the operationalization was adapted to reflect the specific context of transition. Two of our three selected measurements are specific for patient empowerment covering the field of health care (PAM) and transition (TCS) [12].

There are three advantages to using SEM in this study to identify PE, because it (a) allows a full-information analysis and interpretation, (b) enables the observation of changes over time, and (c) provides better methods for the treatment of missing data [32,33]. In particular, identifying and observing changes in PE over time is useful, because this fully views the definition of empowerment as a process [12,14]. One to six months after the intervention, the developed PE is significantly higher than the baseline measurement of the considered components. We conclude that the positive effects of the intervention were stable and the participated adolescents developed empowerment. The adolescents developed adequate knowledge, skills, understanding, and motivation to deal with their chronic conditions and their transition process.

We furthermore extended the model of PE in order to analyze the effect of the intervention, and to determine whether age had an influence on the association between group affiliation (intervention vs. control group) and the development of PE. The aim of the intervention was to investigate whether a higher level of PE in adolescents with chronic conditions was associated with a more active role in managing their own health care and a higher ability to manage their own transition [4,12,14,34]. The patient education program was designed to include different chronic conditions; therefore, it followed a generic approach in order to educate adolescents with a broad variety of conditions within the same program. It was predicted that the participation to the intervention group leads to a higher level of empowerment compared to the control group. The group affiliation loaded positively on the latent variable PE, meaning that the intervention group developed a higher level of patient empowerment than the control group. Consistent with Aujoulat et al. [15], the patient education program was designed to support adolescents with chronic conditions to empower them in different aspects of their life, for example, having a greater sense of self-efficacy, participating more actively in their decision-making processes and having the knowledge of specific aspects of their own health care. Higher patient empowerment is associated with better health-related outcomes [35].

Although we had predicted an effect of age on the development of empowerment, the results showed no significant influence on the association of group affiliation and patient empowerment. Since the results did not indicate any differences between intervening at a younger or an older age, we conclude that the intervention can be offered to adolescents with chronic conditions of varying ages.

### Limitations

The missing values of the LSDm of GSE negatively affected the SRMR value. In the future, all relevant components must be assessed at all measure points in order to improve the fit of the model. This study only considered two influence factors (group affiliation and age); future work should include other predictors such as duration, chronic condition, socio-economic status or sex. Especially, potential confounders regarding the inconsistent follow-up range of 1 to 6 months has to be assessed and included in the future analyses. Wisk et al. [36] found in a retrospective cohort study that girls have an earlier transfer to adult-focused primary care than boys. Regarding the specification of empowerment models, recent studies have used different approaches: Peterson [37] suggested a formative measurement perspective by conceptualizing empowerment as a higher-order multidimensional construct formed by its dimensions, while the study of Miguel et al [38] showed that the reflective measurement model of empowerment was superior to a formative one. These findings suggest that more research on empowerment theory is required to test and develop further models and conceptualizations of empowerment.

## Conclusions

The operationalization of a conceptual model of patient empowerment was successful. The construct is utilizable in the specific context of transition, the identified components are responsive to the influence of a patient education program and can be used for a broad age span in a clinical or ambulant context. Adolescents with chronic conditions have benefited from a higher empowerment in their ability to manage their own health care.

## List of abbreviations

LDSm: Latent Difference Score model
GSE: General Self-Efficacy
TCS: Transition Competence Scale
PAM: Patient Activation Measure
PE: Patient Empowerment
SEM: Structural Equation Modeling
FIML: Full Information Maximum Likelihood
RMSEA: Root Mean Square Error of Approximation
CFI: Comparative Fit Index
SRMR: Standardized Root Mean Residual

## References

[1] Stam H, Hartman EE, Deurloo JA, Groothoff J, Grootenhuis MA. Young Adult Patients with a History of Pediatric Disease: Impact on Course of Life and Transition into Adulthood. J Adolesc Heal. 2006;39: 4–13. 10.1016/j.jadohealth.2005.03.011 16781955

[2] Berg CA, Butner JE, Butler JM, King PS, Hughes AE, Wiebe DJ. Parental Persuasive Strategies in the Face of Daily Problems in Adolescent Type 1 Diabetes Management. 2013;32: 719–728. 10.1037/a0029427 22888825 PMC8530460

[3] Bal MI, Sattoe JNT, Roelofs PDDM, Bal R, van Staa A, Miedema HS. Exploring effectiveness and effective components of self-management interventions for young people with chronic physical conditions: A systematic review. Patient Educ Couns. 2016;99: 1293–1309. 10.1016/j.pec.2016.02.012 26954345

[4] Schmidt S, Herrmann-Garitz C, Bomba F, Thyen U. A multicenter prospective quasi-experimental study on the impact of a transition-oriented generic patient education program on health service participation and quality of life in adolescents and young adults. Patient Educ Couns. 2016;99: 421–428. 10.1016/j.pec.2015.10.024 26597543

[5] Zhou H, Roberts P, Dhaliwal S, Della P. Transitioning adolescent and young adults with chronic disease and / or disabilities from paediatric to adult care services–an integrative review. 2016; 3113–3130. 10.1111/jocn.13326 27145890 PMC5096007

[6] Yassaee A, Hale D, Armitage A, Viner R. The Impact of Age of Transfer on Outcomes in the Transition From Pediatric to Adult Health Systems: A Systematic Review of Reviews. Journal of Adolescent Health. 2019 pp. 709–720. 10.1016/j.jadohealth.2018.11.023 30833120

[7] de Beaufort C, Jarosz-Chobot P, Frank M, de Bart J, Deja G. Transition from pediatric to adult diabetes care: smooth or slippery? Pediatr Diabetes. 2010;11: 24–27. 10.1111/j.1399-5448.2009.00524.x 20015124

[8] Gilliam PP, Ellen JM, Leonard L, Kinsman S, Jevitt CM, Straub DM. Transition of Adolescents With HIV to Adult Care: Characteristics and Current Practices of the Adolescent Trials Network for HIV/AIDS Interventions. J Assoc Nurses AIDS Care. 2011;22: 283–294. 10.1016/j.jana.2010.04.003 20541443 PMC3315706

[9] Sullivan-oliveira JO, Fernandes SM. Transition of Pediatric Patients to Adult Care: An Analysis of Provider Perceptions Across Discipline and Role. 2014;40: 2014. 25134224

[10] de Silva PSA, Fishman LN. Transition of the Patient with IBD from Pediatric toAdult Care—An Assessment of Current Evidence. Inflamm Bowel Dis. 2014;20: 1458–1464. 10.1097/MIB.0000000000000045 24846721

[11] While A, Forbes A, Ullman R, Lewis S, Mathes L, Griffiths P. Good practices that address continuity during transition from child to adult care: synthesis of the evidence. Child Care Health Dev. 2004;30: 439–452. 10.1111/j.1365-2214.2004.00440.x 15320921

[12] Fumagalli LP, Radaelli G, Lettieri E, Bertele P, Masella C. Patient Empowerment and its neighbours: Clarifying the boundaries and their mutual relationships. Health Policy (New York). 2015;119: 384–394. 10.1016/j.healthpol.2014.10.017 25467286

[13] McAllister M, Dunn G, Payne K, Davies L, Todd C. Patient empowerment: The need to consider it as a measurable patient-reported outcome for chronic conditions. BMC Health Serv Res. 2012;12: 1 10.1186/1472-6963-12-1 22694747 PMC3457855

[14] Zimmerman M a. Psychological Empowerment: Issue and Illustrations. Am J Community Psychol. 1995;5: 581. 10.1007/BF02506983 8851341

[15] Aujoulat I, d’Hoore W, Deccache A. Patient empowerment in theory and practice: Polysemy or cacophony? Patient Educ Couns. 2007;66: 13–20. 10.1016/j.pec.2006.09.008 17084059

[16] Schwarzer R., & Jerusalem M. General Self-efficacy Scale. Meas Heal Psychol A user’s portfolio Causal Control beliefs. 1995; 35–37. 10.1037/t00393-000

[17] Hibbard JH, Mahoney ER, Stockard J, Tusler M. Development and Testing of a Short Form of the Patient Activation Measure. Health Serv Res. 2005;40: 1918–1930. 10.1111/j.1475-6773.2005.00438.x 16336556 PMC1361231

[18] McArdle. Dynamic but structural equation modeling of repeated measures data. Handbook of multivariate experimental psychology. 1988 pp. 561–614. 10.1007/978-1-4613-0893-5_17

[19] Bomba F, Herrmann-Garitz C, Schmidt J, Schmidt S, Thyen U. An assessment of the experiences and needs of adolescents with chronic conditions in transitional care: a qualitative study to develop a patient education programme. Health Soc Care Community. 2017;25: 652–666. 10.1111/hsc.12356 28173635

[20] Ernst G, Menrath I, Lange K, Eisemann N, Staab D, Thyen U, et al Development and evaluation of a generic education program for chronic diseases in childhood. Patient Educ Couns. 2017; 1–8. 10.1016/j.pec.2016.11.012 28109650

[21] Menrath I, Ernst G, Szczepanski R, Lange K, Bomba F, Staab D, et al Effectiveness of a generic transition-oriented patient education program in a multicenter, prospective and controlled study. J Transit Med. 2018; 10.1515/jtm-2018-0001

[22] Schmidt S, Herrmann-Garitz C, Bomba F, Thyen U. A multicenter prospective quasi-experimental study on the impact of a transition-oriented generic patient education program on health service participation and quality of life in adolescents and young adults. Patient Educ Couns. 2016;99: 421–428. 10.1016/j.pec.2015.10.024 26597543

[23] Worthington RL, Whittaker TA. Scale development research: A content analysis and recommendations for best practices. Couns Psychol. 2006;34: 806–838. 10.1177/001100006288127

[24] Herrmann-Garitz C, Muehlan H, Bomba F, Thyen U, Schmidt S. Konzeption und Erfassung der gesundheitsbezogenen Transitionskompetenz von Jugendlichen mit chronischen Erkrankungen [Conception and Measurement of Health-related Transition Competence for Adolescents with chronic condition]. Das Gesundheitswes. 2015;79: 491–496. 10.1055/s-0035-1549986 26270040

[25] Bomba F, Markwart H, Mühlan H, Menrath I, Ernst G, Thyen U, et al Adaptation and validation of the German Patient Activation Measure for adolescents with chronic conditions in transitional care: PAM®13 for Adolescents. Res Nurs Heal. 2018;41: 78–87. 10.1002/nur.21831 29266283

[26] Matsunaga M. Item Parceling in Structural Equation Modeling: A Primer. Commun Methods Meas. 2008;2: 260–293. 10.1080/19312450802458935

[27] Muthén L, Muthén B. Mplus user’s guide (7th ed.). Los Angeles. 2014.

[28] Bollen KA, Long JS. Testing structural equation models [Internet]. Testing structural equation models. 1993 Available: http://www.worldcat.org/title/testing-structural-equation-models/oclc/26856260&referer=brief_results

[29] Rappaport Julian, Seidman E. Handbook of Community Psychology. Psychiatr Serv. 2000;52: 1538-a–1539. 10.1176/appi.ps.52.11.1538-a

[30] Mora MA, Sparud-Lundin C, Bratt E-L, Moons P. Empowering Young Persons During the Transition to Adulthood. In: Betz CL, Coyne IT, editors. Transition from Pediatric to Adult Healthcare Services for Adolescents and Young Adults with Long-term Conditions. Cham: Springer International Publishing; 2020 10.1007/978-3-030-23384-6

[31] Holden DJ, Evans WD, Hinnant LW, Messeri P. Modeling Psychological Empowerment Among Youth Involved in Local Tobacco Control Efforts. Heal Educ Behav. 2005;32: 264–278. 10.1177/1090198104272336 15749971

[32] Jaccard J, Wan CK. LISREL approaches to interaction effects in multiple regression [Internet]. Quantitative Applications in the Social Sciences. 1996 10.2307/1271188

[33] Tomarken AJ, Waller NG. Structural Equation Modeling: Strengths, Limitations, and Misconceptions. Annu Rev Clin Psychol. 2005;1: 31–65. 10.1146/annurev.clinpsy.1.102803.144239 17716081

[34] Christens BD, Peterson NA. The Role of Empowerment in Youth Development: A Study of Sociopolitical Control as Mediator of Ecological Systems’ Influence on Developmental Outcomes. J Youth Adolesc. 2012;41: 623–635. 10.1007/s10964-011-9724-9 22038436

[35] Geerlings RPJ, Aldenkamp AP, Gottmer-Welschen LMC, van Staa AL, de Louw AJA. Long-term effects of a multidisciplinary transition intervention from paediatric to adult care in patients with epilepsy. Seizure. 2016;38: 46–53. 10.1016/j.seizure.2016.04.004 27131211

[36] Wisk LE, Finkelstein JA, Sawicki GS, Lakoma M, Toomey SL, Schuster MA, et al Predictors of Timing of Transfer From Pediatric- to Adult-Focused Primary Care. JAMA Pediatr. 2015;169: e150951 10.1001/jamapediatrics.2015.0951 26030515 PMC4862601

[37] Peterson NA. Empowerment Theory: Clarifying the Nature of Higher-Order Multidimensional Constructs. Am J Community Psychol. 2014;53: 96–108. 10.1007/s10464-013-9624-0 24420068

[38] Miguel MC, Ornelas JH, Maroco JP. Defining psychological empowerment construct: Analysis of three empowerment scales. J Community Psychol. 2015;43: 900–919. 10.1002/jcop.21721

